# Common Mechanisms of Developmental Reprogramming in Plants—Lessons From Regeneration, Symbiosis, and Parasitism

**DOI:** 10.3389/fpls.2020.01084

**Published:** 2020-07-16

**Authors:** Yasunori Ichihashi, Tsuneo Hakoyama, Akira Iwase, Ken Shirasu, Keiko Sugimoto, Makoto Hayashi

**Affiliations:** ^1^RIKEN BioResource Research Center, Tsukuba, Japan; ^2^RIKEN Center for Sustainable Resource Science, Yokohama, Japan

**Keywords:** reprogrammed development, lateral root development, parasitism, regeneration, symbiosis

## Abstract

Most plants are exquisitely sensitive to their environment and adapt by reprogramming post-embryonic development. The systematic understanding of molecular mechanisms regulating developmental reprogramming has been underexplored because abiotic and biotic stimuli that lead to reprogramming of post-embryonic development vary and the outcomes are highly species-specific. In this review, we discuss the diversity and similarities of developmental reprogramming processes by summarizing recent key findings in reprogrammed development: plant regeneration, nodule organogenesis in symbiosis, and haustorial formation in parasitism. We highlight the potentially shared molecular mechanisms across the different developmental programs, especially a core network module mediated by the AUXIN RESPONSIVE FACTOR (ARF) and the LATERAL ORGAN BOUNDARIES DOMAIN (LBD) family of transcription factors. This allows us to propose a new holistic concept that will provide insights into the nature of plant development, catalyzing the fusion of subdisciplines in plant developmental biology.

## Introduction

Plants and animals are separated by about 1.5 billion years of evolutionary history and have independently evolved multicellular organizations. Animal development is largely buffered against environmental perturbations, and embryogenesis determines the body plan. On the other hand, plants possess a high degree of developmental plasticity to generate various types of new tissues or organs in response to external stimuli, and they adapt to their environment by altering the course of their post-embryonic development. This developmental plasticity is often achieved through cell identity transitions, or cellular reprogramming, which convert one specific cell type to another (Efroni, 2018; Sugimoto et al., 2018). A typical example of such reprogramming of developmental fate (hereafter, developmental reprogramming) is plant regeneration, which is a biological process wherein a part of a plant is restructured in response to various stimuli. Plant regeneration is often seen after wounding and pathogen infection, and an unorganized cell mass called a callus covers the wound site to repair the tissue (Ikeuchi et al., 2013). Developmental reprogramming is observed not only in response to abiotic environmental stresses but also in plant-microbe and plant-plant interactions. For instance, nodule organogenesis in roots triggered by nitrogen-fixing bacteria is a typical aspect of plant-microbe interactions that accompany *de novo* post-embryonic development. When starved of nitrogen, some plants can establish symbiosis with bacteria, such as rhizobia of Proteobacteria, which eventually fix dinitrogen in the air into ammonia in root nodules to be utilized by host plants (Udvardi and Poole, 2013). The developmental processes are fairly complex—infection of bacteria from the epidermis, concomitant with nodule organogenesis in the cortex, endodermis, and pericycle. Infected bacteria are delivered inside the roots and incorporated into host plant cells in the developing nodules. The nodule is thought to be an organ that offers bacteria a place for effective nitrogen fixation, supplying optimal conditions for such processes as carbon assimilation and oxygen concentration. In addition to plant-microbe interactions, developmental reprogramming mediates the interaction between parasitic plants and their host plants. Parasitic plants infect host plants to obtain nutrients and water. They develop a unique multi-cellular organ called a haustorium that forms when host-derived signals are detected. The haustoria invade the host stem or root and connect the vascular systems of the host and parasite, allowing exchange of various materials, including water, nutrients, proteins, nucleotides, pathogens, and retrotransposons between the host and the parasite (Ichihashi et al., 2015; Yoshida et al., 2016).

**Figure 1:**
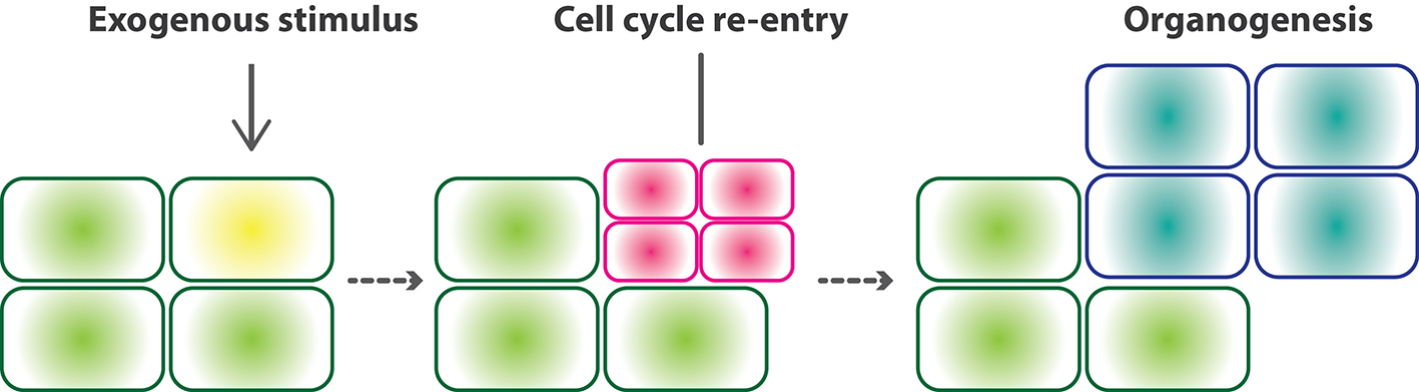
Core concept of developmental reprogramming. Developmental reprogramming, developmental plasticity, and cellular reprogramming include various types of reprogrammed development. All types of reprogrammed development share similar cellular events. Exogenous stimuli triggers differentiated cells to re-enter the cell cycle. Cell proliferation following cell differentiation forms a new organ with different cell types from those of original mother cells.

Although these processes of reprogrammed development serve considerably different physiological functions to meet their distinct adaptive strategies, they are similar at the cellular level, namely, responding to the environmental signals that trigger cell cycle re-entry to generate *de novo* organogenesis (**Figure 1**). Here, we summarize recent findings on the development and gene regulation of regeneration, nodule organogenesis in symbiosis, and haustorial formation in parasitism. This allows us to unveil the deep homology in which gene network modules involved in lateral root development have been co-opted into cellular reprogramming in the modified developmental processes. Combining these, we discuss the generality of developmental reprogramming processes to shed light on the uniqueness of plant development.

### Cellular Features in Reprogrammed Development

We briefly describe how cells in a differentiated organ are transformed into new organs during the course of plant regeneration, nodule organogenesis, and haustorial formation. Using information assembled from different species, we discuss common features of the cellular and morphological framework of reprogramming.

#### Plant Regeneration

**Figure 2:**
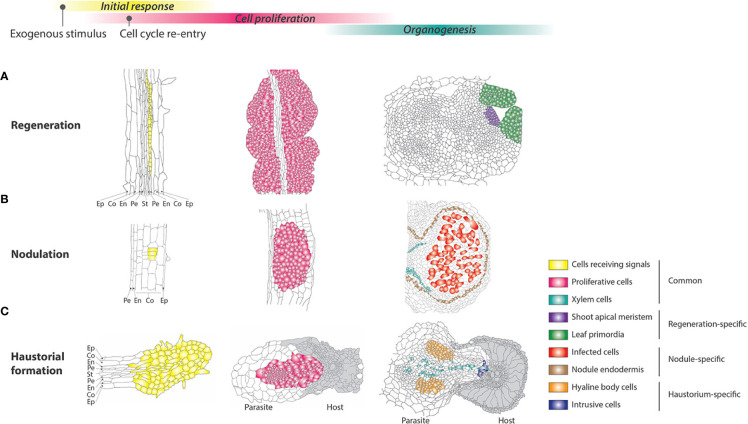
Comparison of cellular and developmental processes among different responses in reprogrammed development. **(A–C)** Schematic illustrations of shoot meristem regeneration from callus, nodulation, and haustorial formation at the cellular level. The cells receiving external signals and actively proliferating are highlighted in yellow and red, respectively. As differentiated cells, xylem cells formed *de novo* and other species-specific differentiated cells are highlighted with green and different color codes, respectively. The epidermis (Ep), cortex (Co), endodermis (En), pericycle (Pe), and stele (St) are also shown. **(A)** When *Arabidopsis* root explants are incubated on auxin-rich callus-inducing medium (CIM), the pericycle cells in the explants respond to the external signals to undergo cell division to form calli. After transfer to shoot-inducing medium (SIM), the callus forms the shoot apical meristem. **(B)** Part of the root cortical cells of legumes enters mitotic activity, forming a cluster of dividing cells, resulting in a nodule. In the mature nodule, the connection of peripheral vascular bundles to the main root vasculature as well as nodule parenchyma, endodermis, and cortex are differentiated. The central region of the nodule maintains large infected cells. **(C)** Parasite *Striga* root perceiving host-derived signals shows swelling along the root and activates cell proliferation to form a haustorium. The epidermis cells differentiate into intrusive cells, and after reaching the host xylem, some of them turn into xylem cells. A cluster of secondary parenchyma cells, called the hyaline body, is also differentiated around the xylem strand. Host tissues are shown in grey.

Regeneration is widely observed in the plant kingdom, from algae to angiosperms (Ikeuchi et al., 2016). Regeneration of seed plants is classified into two modes, initiating either from pre-existing stem cells or from newly formed stem cells. Suppression of apical dominance, which releases the axillary meristems from dormancy, is a clear example of regeneration from pre-existing stem cells (Burian et al., 2016). The latter case often occurs after callus formation; this cell mass can exert pluripotency/totipotency, since shoot, root, tracheary elements, and somatic embryos are generated from it (**Figure 2A**) (White, 1939; Steward et al., 1958; Nagata and Takebe, 1971). The balance between two phytohormones, auxin and cytokinin, is critical for callus induction and the determination of regeneration from it (Skoog and Miller, 1957). Generally, a higher auxin/cytokinin ratio induces root regeneration from callus, while a low ratio induces shoot regeneration. The appropriate balance of phytohormones maintains callus cell proliferation without any regeneration. In the case of *Arabidopsis thaliana* shoot regeneration from root explants, callus is often induced by auxin-rich callus-inducing medium (CIM), where pericycle cells adjacent to the xylem poles lose the expression of a pericycle cell-specific marker within 2–3 days and become a major contributor to cell mass (Valvekens et al., 1988; Che et al., 2007; Atta et al., 2009). Culturing on cytokinin-rich shoot-inducing medium (SIM) after a few days of CIM treatment can trigger shoot meristem formation/organogenesis in the callus (Meng et al., 2017; Wang et al., 2017; Zhang et al., 2017). In addition to the two phytohormones, wounding is another key stimulus for cellular reprogramming, since unwounded root tissue cannot induce shoots even after the two-step treatments of CIM and SIM (Iwase et al., 2011a; Iwase et al., 2015). Excised gametophore leaf cells from moss *Physcomitrella patens* are reprogrammed into chloronema apical stem cells and restart cell division (Ishikawa et al., 2011). Similarly, ablated hypocotyl tissues from Arabidopsis can form callus at the wound site even without exogenous phytohormones (Iwase et al., 2011a). These observations indicate that wounding renders differentiated cells pluripotent and allows to re-enter the cell cycle. The majority of Arabidopsis cells in differentiated tissues are endoreduplicated (Inzé and De Veylder, 2006), but xylem-adjacent pericycle cells are thought to remain diploid, which likely enables the pericycle cells to maintain high proliferation ability (Atta et al., 2009). However, fully differentiated cells such as cortex, epidermal, and endodermal cells can also initiate callus formation and regeneration (Ikeuchi et al., 2016), implying that distinct mechanisms underpin cell cycle re-entry depending on cell type. Cytokinin-responsive D-type cyclins are important cell cycle regulators for cellular reprogramming, since their loss-of-function mutants show reduction in callus formation and shoot organogenesis in Arabidopsis (Dewitte et al., 2007; Ikeuchi et al., 2017). Reactivation of the cell cycle induced by stress can generally start within a few days during the process of regeneration, indicating that cell fate should be changed within this period to execute the program in cell cycle re-entry. Interestingly, nuclear enlargement was detected before cell cycle re-entry in the regeneration process. For example, single mesophyll protoplasts in the regeneration process show larger nuclei compared to those in original differentiated cells (Zhao et al., 2001).

#### Nodule Organogenesis in Symbiosis

Only a limited number of plant species establish root nodule symbiosis with bacteria. The most common is the legume family in the order Fabales, in which the majority of species can develop nodules, whereas nodulation is much rarer in orders Cucurbitales, Fagales, and Rosales (Doyle, 2011). Since they are taxonomically monophyletic, it has been proposed that there was a common ancestor that acquired the ability for this symbiosis (Soltis et al., 1995). Plants that associate with *Frankia* spp. of Actinobacteria to develop nodules are called actinorhizal plants, in contrast to legumes that associate with rhizobia. Ontogenically, nodules developed in actinorhizal plants are of pericycle origin, whereas those in legumes originate from the cortex (Svistoonoff et al., 2014). Differences are also seen in the arrangement of vasculature; the former develops a central vascular bundle, as in the case of lateral roots, whereas the latter shows several peripheral vascular bundles. Nodule vasculature is important for providing bacteria inside host cells with a carbon source and, in return, translocating assimilated nitrogen compounds to host plants (**Figure 2B**). Nodule organogenesis is associated with cell cycle-related genes (Foucher and Kondorosi, 2000). Notably, the earliest expression of B-type cyclin genes is restricted to the foci where the nodule primordium is anticipated, indicating that the initial activation of nodule organogenesis triggers cortical cells in G2 to re-enter the cell cycle. In accordance with this, there are substantial changes in subcellular components. Endoplasmic microtubules become centrally and anticlinally organized, with cytoplasmic rearrangement, which aligns an infecting epidermal cell to the cortical cell layers where the cell cycle is activated (Timmers et al., 1999). Nuclei in the cortical cells, ellipsoid in the differentiated cells, start to migrate to the center of the cells, and become swollen. This pre-infection re-organization of the cell architecture is also observed in association with arbuscular mycorrhizal fungi, which is widespread in land plants and is a much more ancient symbiosis (Bonfante and Genre, 2008). As in the case of plant regeneration, the two major phytohormones, auxin and cytokinin, play pivotal roles in nodule organogenesis. Application of auxin transport inhibitors such as NPA or TIBA can induce nodule structures in the absence of bacteria (Hirsch et al., 1989). Based on circumstantial evidence, it is interpreted that a local and transient decrease in auxin concentration is involved in the initial stage of nodule organogenesis, possibly under the influence of cytokinin, but later, auxin accumulates in the nodule primordium to maintain meristematic activities (Oldroyd et al., 2011). The role of cytokinin, in contrast, is a positive regulator of nodule organogenesis. A gain-of-function mutant of the cytokinin receptor, or even application of cytokinin, can induce nodule structures in the absence of bacteria, whereas loss of the receptor severely compromises nodule organogenesis (Miri et al., 2016).

#### Haustorial Formation in Parasitism

Parasitic plants include about 4,000 species, and have evolved independently more than 11 times during the course of evolution (Barkman et al., 2007). They show various parasitic modes: some parasitic plants infect the stem, while others infect the root of host plants; some parasitic plants are facultative, others are obligate, and some parasitic plants still retain chlorophyll, while others have lost it. Combinations of these features create great diversity within parasitic plants. However, all parasitic plants develop a multicellular organ, a haustorium, which is stated to be “the essence of parasitism” (**Figure 2C**) (Kuijt, 1969). The haustoria in the parasitic Orobanchaceae and Convolvulaceae have been well characterized and classified into two distinct types: terminal haustoria and lateral haustoria. The development of a haustorium usually starts a few hours after exposure to the host roots or host root exudates (Baird and Riopel, 1984; Bandaranayake and Yoder, 2013). 2,6-dimethoxy-*p*-benzoquinone (DMBQ) and its structural analogs are characterized as host-derived signals that originate from cell wall lignin through the oxidation of syringic acid and a phenolic acid (Chang and Lynn, 1986; Yoshida et al., 2016; Cui et al., 2018; Wada et al., 2019). In the case of *Agalinis purpurea*, which is a facultative hemi-parasite, after 6 h on these host-derived signals, the inner cortex cells are vacuolarized and radially enlarged, leading to lateral swelling along the root; after 12 h epidermal cells divide anticlinally to establish a group of densely cytoplasmic cells at the apex of the haustorium; periclinal cell division begins from the innermost cortex and subsequently progresses to the other cortex and pericycle layers (Baird and Riopel, 1984; Wakatake et al., 2018). This observation is consistent with that of another parasite, *Phtheirospermum japonicum*: A cell division marker such as B-type cyclin was expressed in all cell layers in the haustorium within 24 h of exposure to haustorium-inducing chemicals (Ishida et al., 2011; Wakatake et al., 2018). After contact with the host, the epidermis cells at the haustorium apex become densely protoplasmic with enlarged nuclei, followed by rapid elongation of the cells, which are often called intrusive cells or palisade cells (Musselman and Dickison, 1975; Neumann et al., 1998). In addition, haustorial hairs differentiated from epidermal cells support the physical connection with the host plants (Cui et al., 2016). The elongated intrusive cells use enzymatic activity to penetrate between the host cortical cells towards the host vasculature (Neumann et al., 1999). After reaching the host xylems, the intrusive cells grow predominantly through pit membranes into the host xylem element, and turn into vessel elements (Dörr, 1997). Subsequently, cells at the center of the haustorium differentiate into vessels or tracheary elements to form a xylem bridge, establishing host-parasite xylem continuity (Neumann et al., 1998). In addition to this xylem continuity, some obligate parasites in the Orobanchaceae, including *Orobanche crenata* and *Alectra vogelii*, and Convolvulaceae develop phloem cells inside the haustorium to connect to the host vascular system with interspecific plasmodesmata (Dörr et al., 1979; Dörr, 1990; Dörr and Kollmann, 1995). Current clonal analysis combined with molecular markers clearly showed cellular reprogramming in the haustorium, where cell lineages derived from cortex and epidermal cells changed their identity to become procambium-like cells and intrusive cells, respectively (Wakatake et al., 2018). As in the case of plant regeneration and nodule organogenesis, auxin plays a role in haustorial formation. The auxin-responsive DR5 reporter and *IAA2* promoter demonstrated that auxin accumulation is involved in haustorium initiation in *P. japonicum* and *Triphysaria versicolor* (Tomilov et al., 2005; Ishida et al., 2016; Yoshida et al., 2016). In addition, local auxin accumulation is crucial for haustorial development in parasitic plants because exogenous application of auxin increases the number of haustoria, whereas disturbing auxin flux decreases the number of haustoria in *T. versicolor* and *Phelipanche aegyptiaca* (Tomilov et al., 2005; Bar-Nun et al., 2008; Wakatake et al., 2020). Local auxin biosynthesis regulated by the *YUCCA* gene expressed in the epidermal cells near the contact site plays a key role in haustorial formation in *P. japonicum* (Ishida et al., 2016). Interestingly, cytokinins derived from *P. japonicum* as a mobile signal induced morphological changes in their host roots (Spallek et al., 2017).

Taken together, the different modes of reprogrammed development mentioned above share similar cellular events in which the cells in differentiated tissues start to respond to exogenous stimuli with morphologically visible changes. Subsequently, these cells re-enter the cell cycle to induce multicellular organs with species-specific cell differentiation. Of these various series of differentiation programs, *de novo* xylem formation is conserved across responses, suggesting that continuity from the xylem to the central vasculature is required as a lifeline for the new organs. Notably, in response to environmental cues, front-line cells, such as host plant cells adjacent to infecting bacterial cells, parasitic plant cells adjacent to host plant cells, and isolated protoplast cells, show enlargement of nuclei before starting cell division or differentiation. The enlargement of nuclei is also known to be one of the typical cytological changes in mammalian cell reprogramming, in which the differentiated somatic nuclei transferred into enucleated eggs are reprogrammed to acquire totipotent status (Gao et al., 2007). Given that heterochromatin in differentiated somatic cells turns into an euchromatic state along with enlargement of nuclei, chromatin re-arrangement and euchromatic status might be a common intracellular event in developmental reprogramming to allow changes in the expression of genes leading to the activation of new gene regulatory networks.

### Evolutionary Origin of Developmental Reprogramming

Developmental processes are underpinned by dynamic transcriptional regulation. Current molecular genetics and gene expression approaches have revealed the gene regulatory networks behind various developmental processes. In this section, we provide an overview of studies on the molecular genetics of reprogrammed development.

In Arabidopsis, two different molecular mechanisms that control callus formation are well characterized: one is wound-induced and the other is the auxin-induced pathway (Sugimoto et al., 2010; Iwase et al., 2011a; Ikeuchi et al., 2016; Ikeuchi et al., 2019). Callus formation from wound sites in phytohormone-free conditions is specifically regulated by AP2/ERF transcription factors, *WOUND INDUCED DEDIFFERENTIATION* (*WIND*) genes (Iwase et al., 2011a; Iwase et al., 2011b). In contrast, callus formation from unwounded parts of explants cultured on CIM is regulated by the auxin-induced pathway, which is shared with the lateral root (LR) developmental pathway (Atta et al., 2009; Sugimoto et al., 2010). Detailed histological observations revealed that the calli induced on CIM are not actually a mass of unorganized cells, but rather organized structures similar to the LR primordia irrespective of their origin (Atta et al., 2009). This is also supported by the expression of LR meristem-related molecular markers, namely, *SHORT ROOT*, *SCARECROW*, and *PLETHORA1* transcription factors in the callus (Atta et al., 2009; Sugimoto et al., 2010). Consistently, LR formation-deficient mutants, *aberrant lateral root formation 4*, and *solitary root* (*slr*), fail to form callus from the unwounded site (Sugimoto et al., 2010; Iwase et al., 2011a). In *Arabidopsis* LR development, transcriptional activators of auxin response genes, *AUXIN RESPONSE FACTORs* (*ARFs*), regulate LR initiation and development *via* direct activation of the LATERAL ORGAN BOUNDARIES DOMAIN (LBD) family of transcription factors (Okushima et al., 2007). CIM-induced callus formation is impeded in the *arf7 arf19* double mutant, while *LBD16* gain-of-function complements the callus formation phenotype in the double mutant (Fan et al., 2012). Moreover, overexpression of each of *LBD16, 17, 18, 29* is sufficient to induce callus formation without exogenous phytohormones. A recent study revealed that LBD16 also functions in shoot regeneration from callus (Liu et al., 2018). These results suggest that the LR developmental pathway is recruited into auxin-induced callus formation and *de novo* organogenesis. Given that LR development shows cell cycle re-entry and *de novo* stem cell and xylem formation, which is mediated by cellular reprogramming processes, we propose that the LR developmental pathway with the ARF-LBD module might be one of the conserved pathways controlling diversified developmental reprogramming.

Most of the host-plant genes that are required for the onset of nodule organogenesis are also involved in association with arbuscular mycorrhizal fungi. They are called common symbiosis genes, encoding proteins that constitute early signal transduction events during symbioses (Gutjahr and Parniske, 2013). One of them, CCaMK, plays a pivotal role in nodule organogenesis (Singh and Parniske, 2012). A phosphomimic mutation of CCaMK results in spontaneous nodule organogenesis, indicating that activation of CCaMK is sufficient for nodule organogenesis. Downstream of CCaMK, a transcription factor, *NIN*, is required only for nodule organogenesis but not for arbuscular mycorrhizal symbiosis (Soyano and Hayashi, 2014). Ectopic expression of *NIN* confers nodule structures, and this is at least partly due to activation of NF-Y, a CCAAT-binding transcription factor. In contrast, ectopic expression of NF-Y subunit genes, *NF-YA1* and *NF-YB1*, results in an increase in lateral roots, indicating that a part of genetic programs in nodule organogenesis in legumes is co-opted from lateral root development (Soyano et al., 2013). In line with this, nodules in actinorhizal plants are thought to be modified lateral roots (Svistoonoff et al., 2014). In addition, the expression of root apical meristem associated genes, such as *WOX5* and *PLETHORAs*, indicates that legume nodules also have root identity (Franssen et al., 2015). These facts imply that at least some of the mechanisms required for LR development would be recruited to nodule organogenesis. Recently, it was shown that expression of *LBD16*, a key gene for lateral root development, is regulated by NIN during nodule primordia formation (Schiessl et al., 2019; Soyano et al., 2019), suggesting that the host plants co-opt for key regulatory components of lateral root organogenesis in nodule organogenesis.

In the case of haustorial formation, it has been proposed that haustoria originated by mutation-based modification of roots (Kuijt, 1969). On the other hand, it has been proposed that parasites may have evolved through endophytic association or horizontal gene transfer from microorganisms that could confer parasitic ability because haustoria of parasitic plants are morphologically similar to nodules and crown galls (Atsatt, 1973). The current comparative transcriptome study in Orobanchaceae suggests that gene expression patterns in haustorial tissues are similar to those of roots, probably due to shared functions such as being highly specialized underground organs for nutrient uptake and transfer (Yang et al., 2015). In addition, this study identified similar expression patterns of floral tissue such as pollen in the haustoria, suggesting that the penetration of the haustorium may have co-opted genes from the polarized invasive growth seen in pollen tubes (Yang et al., 2015). In terms of development and cellular morphology, recent transcriptome analyses in *Striga hermonthica* and *Thesium chinense* showed that the formation of haustoria and LR goes through a similar process: auxin accumulates at the initiation sites, cell proliferation is reactivated, and cell walls are dramatically remodeled (Yoshida et al., 2016; Ichihashi et al., 2018; Yoshida et al., 2019). Notably, the pericycle-derived lateral root primordia grow through their own endodermal, cortical, and epidermal layers, while the haustorium grows through the host cell layers, such as the epidermis, cortex, endodermis, and pericycle, to reach the host stele. Thus, haustorial formation would utilize at least part of the mechanisms required for LR development.

**Figure 3:**
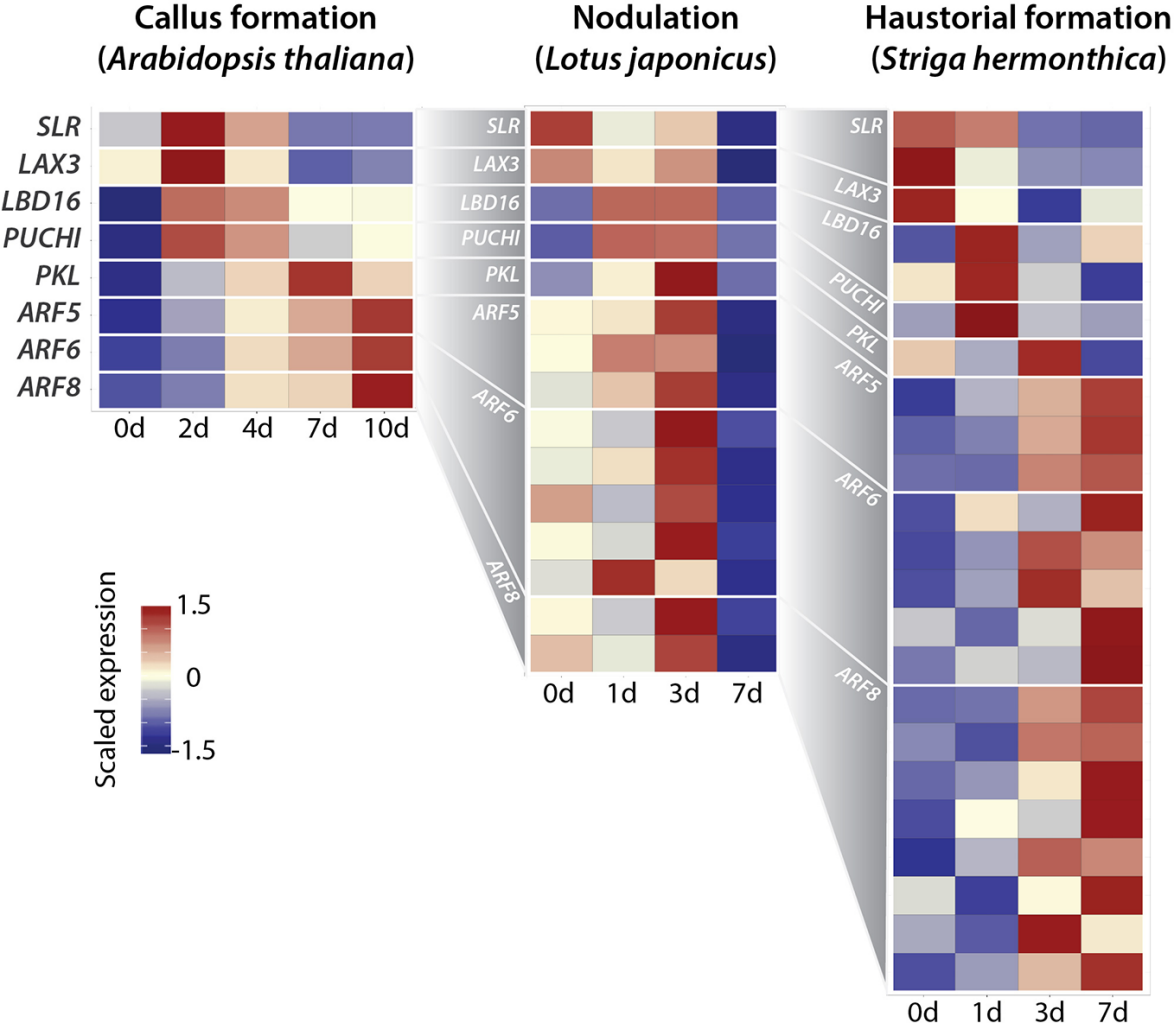
Recruitment of lateral root (LR) developmental pathway into developmental reprogramming. Heat map of scaled gene expression of each transcript for LR developmental genes/orthologs in callus formation (*Arabidopsis thaliana*), nodule organogenesis (*Lotus japonicus*), and haustorial formation (*Striga hermonthica*). LR developmental genes were curated by literatures (Lavenus et al., 2013), and blastx (threshold e-value 1e-10) alignments with *L. japonicus* and *S. hermonthica* transcript sequences against the TAIR10 peptide sequence were performed to determine the best blast-hit gene as their orthologs. Datasets were used from (Che et al., 2006; Høgslund et al., 2009; Yoshida et al., 2019). For callus formation, *A. thaliana* root explants were incubated on an auxin-rich callus-induction medium for the times indicated (0, 2, 4, 7, and 10 days). For nodule organogenesis, *L. japonicus* roots were inoculated with *Mesorhizobium loti* at the times indicated (0, 1, 3, and 7 days). For haustorial formation, rice was infected with *S. hermonthica* seedlings at the times indicated (0, 1, 3, and 7 days). Since several paralogs show different expression patterns from each other in *L. japonicus* and *S. hermonthica*, indicating sub-functionalization in transcriptional regulation, only paralogs with corresponding expression patterns to *A. thaliana* genes are shown.

The above circumstantial evidence led us to test the idea that the LR developmental pathway with the *ARF-LBD* module may be activated during regeneration, nodulation, and haustorium formation (**Figure 3**) by reanalyzing the published transcriptome datasets of CIM-induced callus formation in *Arabidopsis thaliana*, nodule organogenesis in *Lotus japonicus*, haustorial formation in *Striga hermonthica* (Che et al., 2006; Høgslund et al., 2009; Yoshida et al., 2019). In *A. thaliana* LR development, *SLR* and *ARF19* function as modules to regulate the expression of the auxin influx carrier gene *LIKE-AUX 3 (LAX3)* and localized auxin accumulation in the pericycle (Swarup et al., 2008). *ARF19* directly regulates *LBD16*, which positively regulates LR formation (Okushima et al., 2007). *LBD16* is expressed in LR founder cells prior to LR initiation, and expression of the dominant negative form of *LBD16* in LR founder cells completely blocks nuclear migration and LR initiation, indicating that *LBD16* is necessary for LR founder cell polarity acquisition downstream of auxin (Goh et al., 2012). In addition, S*LR-ARF19* regulates *PUCHI*, which locally inhibits cell proliferation to shape LR formation (Hirota et al., 2007). *PICKEL* (*PKL*) is an ATP-dependent chromatin remodeling factor that suppresses LR formation (Fukaki et al., 2006). *ARF5, ARF6*, and *ARF8* modules follow *SLR-ARF19* expression to control LR organogenesis (De Smet et al., 2010; Lavenus et al., 2015). After BLAST searching to identify the orthologous relationships between genes in the datasets from different species, we found the expression of all orthologs of *SLR, LAX3, LBD16, PUCHI, PKL, ARF5, ARF6*, and *ARF8* in all datasets of regeneration, nodule organogenesis, and haustorial formation, except for *ARF19*. Surprisingly, the gene expression patterns across the course of developmental reprogramming processes completely fit the developmental timing seen in the LR development pathway (**Figure 3**). This strongly supports our idea that the mechanism regulating developmental reprogramming shows homology with the recruitment of the *ARF-LBD* module. Given that *LBD* and *ARF* gene evolution can be traced back to aquatic ancestors of land plants, followed by relatively recent lineage-specific expansions on land (Finet et al., 2012; Chanderbali et al., 2015; Kong et al., 2017; Mutte et al., 2018; Martin-Arevalillo et al., 2019), evolution of the *ARF-LBD* module allowed ancestral plants to innovate the developmental reprogramming in response to various types of signals to adapt to diversified land environments. In addition to the possibility of subfunctionalization of *ARF-LBD* module itself during the course of evolution, the *ARF-LBD* module could function only to give potential for a new organ, and cooperate with different modules to specify different organs according to different external stimuli.

## Conclusion

In this review, we summarize studies of plant regeneration, nodule organogenesis in symbiosis, and haustorial formation in parasitism in terms of cellular and molecular aspects of development. Other phenomena, such as nematode-induced root knot formation, grafting between plants, insect/bacteria-induced gall formation as well as novel evolutionary traits that have innovated during the repetitive processes of adaptation against the environment through successive generations, can also be mediated by developmental reprogramming. Our comparative analysis using published gene expression data supports the idea that the auxin-driven *ARF-LBD* module is recruited into the core machinery of reprogrammed developments. Further research using next-generation sequencing and bioinformatics will allow us to reveal the molecular mechanisms in non-model species to provide a greater understanding of the general mechanisms of plant development. Epigenomic alterations, such as histone modification and DNA methylation, have been identified as playing important roles in determining the developmental capabilities of plant stem cells (Ikeuchi et al., 2015a; Ikeuchi et al., 2015b; Ishihara et al., 2019; Sugimoto et al., 2019). Indeed, disruption of histone modifier Polycomb Repressive Complex 2 (PRC2) causes dedifferentiation, callus formation, and somatic embryo formation from fully differentiated root hair cells (Ikeuchi et al., 2015a). The fact that nuclear enlargement and gene expression of the PKL chromatin remodeling factor are conserved in developmental reprogramming, as referred to in this review, is one of the signs that cellular reprogramming with nuclear euchromatin status and epigenomic regulation might be an additional layer of shared molecular mechanisms seen in developmental reprogramming. Relative to animal development, plant development shows high plasticity through cellular reprogramming. Therefore, we believe that answering the following questions centered on developmental reprogramming will provide a generalized paradigm for understanding the nature of plant development:

- What is the extent of conservation of cellular and molecular mechanisms in developmental reprogramming?
- What determines the differences between distinct processes of reprogrammed development?
- Is the *ARF-LBD* module involved in the deep homology of processes of reprogrammed development?
- Is the gene module interchangeable between distinct processes of reprogrammed development?
- Which types of epigenetic modifications regulate developmental reprogramming?
- What is the ancestral function of the *ARF-LBD* module?

## Author Contributions

YI, TH, and AI conceived the topic and YI reanalyzed the published data. All authors contributed to the article and approved the submitted version.

## Funding

This work was supported by the RIKEN Special Postdoctoral Researchers Program; Grant-in-Aid for Young Scientists (B; grant no. 15K18589) from the Ministry of Education, Culture, Sports, Science and Technology, Japan (MEXT); PRESTO, Japan Science and Technology Agency (grant no. JPMJPR15Q2); the Cabinet Office, Government of Japan, Cross-ministerial Strategic Innovation Promotion Program (SIP), “Technologies for Smart Bio-industry and Agriculture”(funding agency: Bio-oriented Technology Research Advancement Institution, NARO) to YI, and MEXT to AI (grant no.18H04849), KSh (grant no. 15H05959), KSu (grant no. 15H05961), and MH (grant no. 17H06472).

## Conflict of Interest

The authors declare that the research was conducted in the absence of any commercial or financial relationships that could be construed as a potential conflict of interest.
